# Survival and HIV-Free Survival Among Children Aged ≤3 Years — Eight Sub-Saharan African Countries, 2015–2017

**DOI:** 10.15585/mmwr.mm6919a3

**Published:** 2020-05-15

**Authors:** Sasi Jonnalagadda, Katharine Yuengling, Elaine Abrams, Paul Stupp, Andrew Voetsch, Monita Patel, Zandi Minisi, Michael Eliya, Ndapewa Hamunime, Anath Rwebembera, Wilford Kirungi, Lloyd Mulenga, Angela Mushavi, Caroline Ryan, Mamorapeli Ts'oeu, Evelyn Kim, Eric J. Dziuban, Kathy Hageman, Jennifer Galbraith, Keith Mweebo, Annie Mwila, Elizabeth Gonese, Hetal Patel, Surbhi Modi, Suzue Saito

**Affiliations:** ^1^CDC; ^2^ICAP at Columbia University, New York, New York; ^3^Eswatini Ministry of Health; ^4^Malawi Ministry of Health; ^5^Namibia Ministry of Health; ^6^Tanzania National AIDS Control Program; ^7^Uganda Ministry of Health; ^8^Zambia Ministry of Health; ^9^Zimbabwe Ministry of Health; ^10^CDC-Eswatini; ^11^CDC-Lesotho; ^12^CDC-Malawi; ^13^CDC-Namibia; ^14^CDC-Tanzania; ^15^CDC-Uganda; ^16^CDC-Zambia; ^17^CDC-Zimbabwe.

Although mother-to-child transmission (MTCT) of human immunodeficiency virus (HIV) is preventable through antiretroviral treatment (ART) during pregnancy and postpartum, the Joint United Nations Programme on HIV/AIDS (UNAIDS) estimates that 160,000 new HIV infections occurred among children in 2018 (*1*). Child survival and HIV-free survival rates* are standard measures of progress toward eliminating MTCT^†^ (*2*). Nationally representative Population-based HIV Impact Assessment (PHIA)^§^ survey data, pooled from eight sub-Saharan African countries^¶^ were used to calculate survival probability among children aged ≤3 years by maternal HIV status during pregnancy and HIV-free survival probability among children aged ≤3 years born to women with HIV infection, stratified by maternal ART** status during pregnancy. Survival probability was significantly lower among children born to women with HIV infection (94.7%) than among those born to women without HIV infection (97.6%). HIV-free survival probability of children born to women with HIV infection differed significantly by the timing of initiation of maternal ART: 93.0% among children whose mothers received ART before pregnancy, 87.8% among those whose mothers initiated ART during pregnancy, and 53.4% among children whose mothers did not receive ART during pregnancy. Focusing on prevention of HIV acquisition and, among women of reproductive age with HIV infection, on early diagnosis of HIV infection and ART initiation when applicable, especially before pregnancy, can improve child survival and HIV-free survival.

Females aged ≥15 years who provided consent^††^ to survey participation answered questions about the most recent pregnancy that resulted in a live birth in the 3 years preceding the interview (i.e., births occurring during 2012–2017, depending on the date of the survey). Questions asked whether any antenatal care was received, timing of HIV testing and HIV status (HIV diagnosis before pregnancy; during pregnancy, labor, or delivery; or did not have a diagnosis of HIV infection), and ART use among mothers with HIV infection (initiated ART before pregnancy; initiated ART during pregnancy, labor, or delivery; or did not receive ART). All mothers provided the child’s date of birth; whether the child was living or deceased, and if deceased, the date of death or age at death; and HIV status of living children. All mothers and a random subsample of children underwent HIV testing in the household using country-specific HIV rapid testing algorithms. Positive rapid test results were confirmed using Geenius HIV-1/2 confirmatory assay (Bio-Rad) (for children aged ≥18 months).^§§^ Infants aged <18 months were screened for HIV exposure using rapid tests; a positive rapid test result was confirmed using total nucleic acid polymerase chain reaction. HIV test results were provided to the participants along with referral to HIV treatment services. Survey protocols for each of the eight countries were reviewed by the CDC Institutional Review Board (IRB), the Columbia University Medical Center IRB, and the IRB in each country.

Children were classified according to maternal report of HIV status during pregnancy to determine survival by maternal HIV status. For the HIV-free survival analysis, children born to mothers with HIV infection were classified according to maternal self-reported ART use during pregnancy. Mothers whose response to HIV status during pregnancy was missing but who had positive test results for HIV (0.8%), were classified as having HIV infection during pregnancy. Mothers with HIV infection who were missing information on ART use during pregnancy (5%) were classified as not having received ART during pregnancy.

HIV status of children in this analysis was determined by HIV testing during the survey (74%) or maternal report for nonsampled children (26%). Date of the child’s HIV diagnosis was based on the survey test date for sampled children and on mothers’ report of first HIV test date with positive results for nonsampled children. Mothers reported the date of death or age at death for children who had died. HIV status of deceased children was not recorded uniformly across surveys and was therefore not included in the analysis. Children without a survey-confirmed HIV status and without mothers’ report were excluded. 

Kaplan-Meier survival analyses were used to estimate overall survival and HIV-free survival probability (*3*). Interview data about mothers’ last pregnancy were used to determine the outcomes of children using the age of the child at the time of events of interest. To estimate survival, children were censored at the age at death or age at time of survey. To estimate HIV-free survival, children of mothers with HIV infection were censored at their age at death, their age at HIV diagnosis, or their age at the time of survey. A sensitivity analysis was conducted to estimate HIV-free survival rates after excluding children currently breastfeeding who were still at risk for HIV infection through breast milk transmission. The analyses were unweighted. Analyses were performed using SAS (version 9.4; SAS Institute) and Stata (version 14.2; StataCorp) statistical software.

Among 36,278 live births, data for the survival analysis were available for 33,863 (93%), including 30,703 (91.0%) children born to mothers without HIV infection, 3,020 (9.0%) born to mothers with HIV infection, and 140 (0.4%) children whose mothers’ HIV status was unknown (Table 1). Among children born to mothers with HIV infection, 108 (3.6%) died; 552 (1.8%) mothers without HIV infection and five (3.6%) mothers with unknown HIV status also died. Cumulative probability of survival up to 3 years among children born to mothers with HIV infection was 94.7% and among children born to mothers without HIV infection was 97.6% (p<0.001) (Figure) (Table 2).

**TABLE 1:** Characteristics of children aged ≤3 years at the time of the Population-based HIV Impact Assessment (PHIA) survey, as determined by maternal report of the most recent pregnancy (N = 33,863) — eight sub-Saharan African countries,* 2012–2017

Characteristic	Eswatini	Lesotho	Malawi	Namibia	Tanzania	Uganda	Zambia	Zimbabwe	Total
**No. of children eligible^†^**	**1,369**	**1,872**	**4,389**	**2,966**	**7,283**	**6,619**	**4,965**	**4,400**	**33,863**
**Child’s age at time of survey (yrs), no. (%)**									
<1	423 (30.9)	605 (32.3)	1,218 (27.8)	998 (33.6)	2,299 (31.6)	2,249 (34.0)	1,542 (31.1)	1,232 (28.0)	**10,566 (31.2)**
1	391 (28.6)	498 (26.6)	1,232 (28.1)	867 (29.2)	2,178 (29.9)	2,034 (30.7)	1,460 (29.4)	1,202 (27.3)	**9,862 (29.1)**
2	340 (24.8)	416 (22.2)	1,101 (25.1)	674 (22.7)	1,699 (23.3)	1,442 (21.8)	1,141 (23.0)	1,124 (25.5)	**7,937 (23.4)**
3	215 (15.7)	353 (18.9)	838 (19.1)	427 (14.4)	1,107 (15.2)	894 (13.5)	822 (16.6)	842 (19.1)	**5,498 (16.2)**
**Sex, no. (%)**									
Female^§^	506 (50.8)	757 (49.2)	2,017 (50.2)	1,117 (50.6)	3,395 (50.6)	2,799 (49.8)	2,259 (50.7)	1,835 (49.2)	**14,685 (50.2)**
Male^§^	490 (49.2)	781 (50.8)	1,997 (49.8)	1,089 (49.4)	3,314 (49.4)	2,822 (50.2)	2,200 (49.3)	1,893 (50.8)	**14,586 (49. 8)**
Unknown^¶^	373 (27.2)	334 (17.8)	375 (8.5)	760 (25.6)	574 (7.9)	998 (15.1)	506 (10.2)	672 (15.3)	**4,592 (13.6)**
**Place of delivery, no. (%)**									
Institution^§^	1,268 (92.6)	1,607 (85.8)	4,123 (93.9)	2,634 (88.8)	5,484 (75.3)	5,159 (77.9)	4,177 (84.1)	3,771 (85.7)	**28,223 (83.3)**
Home^§^	89 (6.5)	245 (13.1)	186 (4.2)	299 (10.1)	1,697 (23.3)	1,269 (19.2)	724 (14.6)	543 (12.3)	**5,052 (14.9)**
Other^§^	12 (0.9)	20 (1.1)	78 (1.8)	31 (1.0)	102 (1.4)	191 (2.9)	63 (1.3)	86 (2.0)	**583 (1.7)**
Unknown^¶^	0 (0.0)	0 (0.0)	2 (0.0)	2 (0.1)	0 (0.0)	0 (0.0)	1 (0.0)	0 (0.0)	**5 (0.0)**
**Residence, no. (%)**									
Urban	303 (22.1)	798 (42.6)	1,482 (33.8)	1,274 (43.0)	2,212 (30.4)	1,569 (23.7)	1,890 (38.1)	1,285 (29.2)	**10,813 (31.9)**
Rural	1,066 (77.9)	1,074 (57.4)	2,907 (66.2)	1,692 (57.0)	5,071 (69.6)	5,050 (76.3)	3,075 (61.9)	3,115 (70.8)	**23,050 (68.1)**
**Ever breastfed, no. (%)**									
Yes^§^	1,248 (92.2)	1,757 (93.9)	4,329 (98.6)	2,876 (97.0)	7,225 (99.2)	6,518 (98.6)	4,887 (98.4)	4,350 (98.9)	**33,190 (98.1)**
No^§^	105 (7.8)	115 (6.1)	58 (1.3)	90 (3.0)	57 (0.8)	94 (1.4)	78 (1.6)	49 (1.1)	**646 (1.9)**
Unknown^¶^	16 (1.2)	0 (0.0)	2 (0.0)	0 (0.0)	1 (0.0)	7 (0.1)	0 (0.0)	1 (0.0)	**27 (0.1)**
**Currently breastfeeding (among ever breastfed), no. (%)**									
Yes^§^	377 (30.2)	606 (34.5)	2,368 (55.5)	1,086 (37.8)	3,558 (49.2)	3,477 (53.5)	2,476 (51.5)	1,757 (40.9)	**15,705 (47.6)**
No^§^	871 (69.8)	1,151 (65.5)	1,896 (44.5)	1,790 (62.2)	3,667 (50.8)	3,019 (46.5)	2,334 (48.5)	2,534 (59.1)	**17,262 (52.4)**
Unknown^¶^	0 (0.0)	0 (0.0)	65 (1.5)	0 (0.0)	0 (0.0)	22 (0.3)	77 (1.6)	59 (1.4)	**223 (0.7)**
Died, no. (%)**	19 (1.4)	52 (2.8)	94 (2.1)	51 (1.7)	146 (2.0)	119 (1.8)	104 (2.1)	80 (1.8)	**665 (2.0)**
**Child’s HIV status, no. (%)**									
HIV-positive^§,††^	14 (1.8)	12 (1.2)	19 (1.0)	5 (0.4)	17 (0.6)	23 (0.6)	19 (0.9)	23 (1.2)	**132 (0.9)**
HIV-negative^§,††^	768 (98.2)	984 (98.8)	1,811 (99.0)	1,266 (99.6)	2,613 (99.4)	3,765 (99.4)	2,098 (99.1)	1,933 (98.8)	**15,238 (99.1)**
Unknown^¶,§§^	587 (42.9)	876 (46.8)	2,559 (58.3)	1,695 (57.1)	4,653 (63.9)	2,831 (42.8)	2,848 (57.4)	2,444 (55.5)	**18,493 (54.6)**
**Mother’s HIV status, no. (%)**									
HIV-positive	441 (32.2)	406 (21.7)	390 (8.9)	398 (13.4)	247 (3.4)	260 (3.9)	388 (7.9)	490 (11.3)	**3,020 (9.0)**
HIV-negative	927 (67.8)	1,463 (78.3)	3,983 (91.1)	2,563 (86.6)	7,028 (96.6)	6,352 (96.1)	4,545 (92.1)	3,842 (88.7)	**30,703 (91.0)**
Not tested	1 (0.1)	3 (0.2)	16 (0.4)	5 (0.2)	8 (0.1)	7 (0.1)	32 (0.6)	68 (1.5)	**140 (0.4)**
**ART status during pregnancy among mothers with HIV infection (N = 2,373 included in HIV-free survival analysis), no. (%)**									
Treated with ART at first antenatal visit^§^	173 (56.5)	142 (45.1)	153 (45.9)	211 (75.6)	75 (44.9)	130 (57.5)	154 (49.2)	214 (49.7)	**1,252 (52.8)**
Newly initiated ART during pregnancy or labor^§^	107 (35.0)	122 (38.7)	152 (45.6)	42 (15.1)	66 (39.5)	65 (28.8)	120 (38.3)	168 (39.0)	**842 (35.5)**
Did not receive ART during pregnancy or labor^§^	26 (8.5)	51 (16.2)	28 (8.4)	26 (9.3)	26 (15.6)	31 (13.7)	39 (12.5)	49 (11.4)	**276 (11.6)**
Unknown^¶^	0 (0.0)	0 (0.0)	0 (0.0)	0 (0.0)	1 (0.6)	0 (0.0)	1 (0.3)	1 (0.2)	**3 (0.1)**

**Abbreviations:** ART = antiretroviral therapy; HIV = human immunodeficiency virus.

* Eswatini, Lesotho, Malawi, Namibia, Tanzania, Uganda, Zambia, and Zimbabwe.

^†^ Eligibility requirements for inclusion in this analysis were that mothers be aged ≥15 years, had a live birth in the most recent delivery within the 3 years preceding the PHIA survey, and had age information on the children.

^§^ Percentages were calculated using only total of children with reported characteristics as the denominator.

^¶^ Percentages were calculated using total of all reported children as the denominator.

** Among 665 children’s deaths, five (9.8%) children were HIV-infected, 23 (3.5%) were not HIV-infected, and for 637 (97.3%), HIV status was not known.

^†† ^As determined by HIV testing conducted during the survey or on mothers’ report of child’s HIV status if child was not sampled for survey HIV testing.

^§§^ Child was not tested for HIV during the survey, and mother’s report of child’s status was missing.

**FIGURE:**
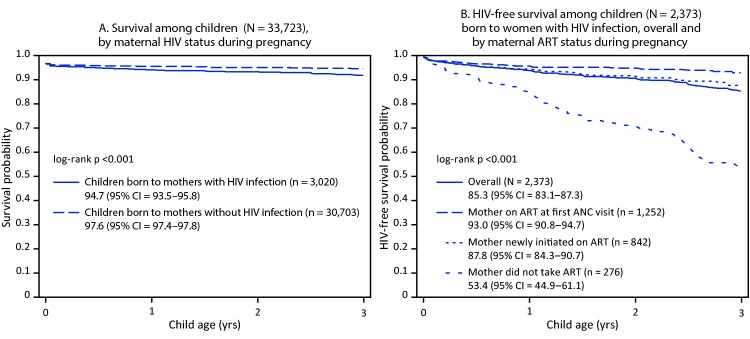
Probability of survival (A)*^,†^ and HIV-free survival (B)^§^ among children aged ≤3 years at the time of the Population-based HIV Impact Assessment (PHIA) survey — eight sub-Saharan African countries,^¶^ 2015–2017 **Abbreviations:** ANC = antenatal care; ART = antiretroviral therapy; CI = confidence interval; HIV = human immunodeficiency virus. * Excludes 140 children out of 33,863 whose mothers had unknown HIV status. ^†^ Excludes 11 deaths among children not exposed to HIV that took place after 3 years. ^§^ Out of 2,373 children born to mothers with HIV infection, three were born to mothers with missing ART use data. ^¶^ Eswatini, Lesotho, Malawi, Namibia, Tanzania, Uganda, Zambia, and Zimbabwe.

**TABLE 2:** Country-specific cumulative probability of survival in children aged ≤3 years at the time of the Population-based HIV Impact Assessment (PHIA) survey — eight sub-Saharan African countries,* 2015–2017

Country	PHIA survey year	All children aged ≤3 years		Children born to mothers with HIV infection		Children born to mothers without HIV infection	
		No.	Survival probability (95% CI)	No.	Survival probability (95% CI)	No.	Survival probability (95% CI)
Eswatini	2016–2017	1,369	97.8 (96.3–98.7)	441	98.2 (95.9–99.2)	927	97.5 (95.5–98.7)
Lesotho	2016–2017	1,872	96.8 (95.7–97.6)	406	93.7 (90.0–96.1)	1,463	97.6 (96.6–98.4)
Malawi	2015–2016	4,389	97.2 (96.5–97.8)	390	90.3 (84.6–94.0)	3,983	97.9 (97.3–98.3)
Namibia	2017	2,966	97.3 (96.4–98.0)	398	97.0 (93.3–98.6)	2,563	97.4 (96.4–98.1)
Tanzania	2016–2017	7,283	97.3 (96.7–97.7)	247	92.8 (87.2–96.0)	7,028	97.4 (96.9–97.9)
Uganda	2016–2017	6,619	97.6 (97.1–98.1)	260	96.9 (91.0–99.0)	6,352	97.6 (97.1–98.1)
Zambia	2016	4,965	96.9 (96.1–97.5)	388	91.8 (86.4–95.1)	4,545	97.3 (96.6–97.9)
Zimbabwe	2015–2016	4,400	97.7 (97.1–98.2)	490	96.6 (94.4–98.0)	3,842	98.1 (97.5–98.5)
**Overall** ^†^	**—**	**33,863**	**97.3 (97.1–97.6)**	**3,020**	**94.7 (93.5–95.8)**	**30,703**	**97.6 (97.4–97.8)**

**Abbreviation:** CI = confidence Interval.

* Eswatini, Lesotho, Malawi, Namibia, Tanzania, Uganda, Zambia, and Zimbabwe.

^†^ HIV status is unknown for mothers of 140 children, who are excluded from the survival analysis by maternal HIV status.

Among the 3,020 children born to mothers with HIV infection, 2,373 (78.6%) had complete HIV data (HIV status and diagnosis date) and death data available and were included in the HIV-free survival analysis. Among these 2,373 children, mothers of 1,252 (52.8%) received ART before pregnancy; 842 (35.5%) initiated ART during pregnancy, labor, or delivery; and 276 (11.6%) did not receive ART during pregnancy, labor, or delivery (Table 1). Overall, 127 (5.4%) of these children had HIV infection, 2,138 (90.1%) did not, and 108 (4.6%) had died.

HIV-free survival probability in children born to mothers with HIV infection was 85.3%. HIV-free survival rates among children whose mothers initiated ART before pregnancy, during pregnancy, and who did not receive ART during pregnancy were 93.0%, 87.8%, and 53.4%, respectively (log-rank p-value <0.001) (Figure). Excluding children who were currently breastfeeding did not alter the HIV-free survival estimates.

## Discussion

The PHIA surveys provide population-level estimates of child survival and HIV-free survival in eight sub-Saharan African countries among children born during 2012–2017, allowing population-level assessment of progress toward elimination of MTCT. The estimated probability of survival of children born to mothers with HIV infection was lower than that of children born to mothers without HIV infection, as has been previously reported (*4*). Previous studies on child mortality by maternal HIV status were conducted before the widespread scale–up of Option B+ (lifelong ART for all pregnant and breastfeeding mothers living with HIV infection regardless of CD4 cell count or clinical stage)^¶¶^ that occurred during 2011–2014 and the 2016 “treat-all” guidance for all persons living with HIV infection (*5*,*6*). Most children included in this analysis were conceived or born before or during the early efforts to scale up adult*** and pediatric^†††^ ART (*6*). The difference in survival probability of children born to mothers with HIV infection and those without HIV infection in the recent birth cohorts^§§§^ appears to be narrowing, which could be the early sign of progress in reducing AIDS-specific morbidity and mortality among adults, potentially conferring survival benefits to children (*7*).

The HIV-free survival rate of 85.3% suggests that substantial gaps remain in improving child survival and eliminating MTCT. HIV-free child survival probability was highest when mothers received ART before pregnancy, compared with survival probability of children whose mothers initiated ART during pregnancy or who did not receive ART during pregnancy, as has been reported in another impact assessment of the prevention of mother-to-child transmission (PMTCT) (*8*). Initiation of ART before pregnancy reduces in utero MTCT of HIV and is associated with postpartum ART retention, which, in turn, reduces the risk for HIV transmission through breastfeeding (*8*,*9*). In this analysis, >90% of mothers with HIV infection received ART during pregnancy, but only approximately 50% initiated ART before pregnancy. These results suggest that achieving MTCT elimination targets will require earlier diagnosis of HIV infection and earlier initiation of ART among women of reproductive age with HIV infection, along with prevention of HIV acquisition among women, prevention of unintended pregnancies, and safe conception planning for mothers living with HIV infection so that they can receive ART and be virally suppressed before pregnancy.^¶¶¶^The difference in HIV-free survival in children determined by maternal ART status during pregnancy was least pronounced in the most recent birth cohort (i.e., children aged ≤1 year at the time of the survey); these children likely benefited most from Option B+ and adult treat-all programs.****

The findings in this report are subject to at least three limitations. First, children in the sample were born during 2012–2017 and received different care depending on HIV treatment standards at the time, which could limit comparability over time. Second, the cross-sectional nature of the data precludes attribution of results to different HIV program effects. Some of the favorable outcomes in children aged ≤1 year could be the consequence of exposure to more effective programs and the shorter duration of observation; however, given that past studies have shown most diagnoses of HIV infection and HIV-associated deaths occurring in the first year of life (*10*), it is more likely to be related to better programs than to shorter observation periods. Finally, mortality was estimated from the most recent live birth during the preceding 3 years; therefore, these mortality estimates are lower than are those from Demographic and Health Surveys, which estimate infant mortality using all deaths during the preceding 5 years.^††††^

Despite considerable scale-up of ART and other PMTCT interventions in sub-Saharan Africa, children born to mothers with HIV infection are still at substantial risk for MTCT of HIV and have lower survival rates than do children born to mothers without HIV infection. In addition to prevention of HIV acquisition among women, HIV programs should focus efforts on early diagnosis of HIV infection and initiation of ART among women of reproductive age with HIV infection, especially before pregnancy, to have the greatest impact in reducing MTCT and reaching child survival goals. Ongoing assessments of survival and HIV-free survival will be needed to determine longer-term effects of improving HIV programs on child health outcomes.

**Summary**

**What is already known about this topic?**

Mother-to-child transmission of human immunodeficiency virus (HIV) is preventable through antiretroviral treatment (ART) during pregnancy and postpartum.

**What is added by this report?**

Among children born to mothers with HIV infection in eight sub-Saharan African countries, HIV-free survival was highest among children whose mothers received ART before pregnancy, compared with those who initiated ART during pregnancy or those who did not receive ART during pregnancy.

**What are the implications for public health practice?**

In addition to prevention of HIV acquisition, national programs should prioritize early diagnosis of HIV infection and ART initiation among women of reproductive age before pregnancy to reduce mother-to-child transmission of HIV and improve child survival rates.

## Footnotes

* For this analysis, HIV-free survival was defined as the child being alive and HIV-negative at the time of the survey, as determined by either the HIV testing conducted during the survey or the maternal report of the child’s HIV status

† Elimination of new HIV infections among children.

§ https://phia.icap.columbia.edu/.

¶ Eswatini, Lesotho, Malawi, Namibia, Tanzania, Uganda, Zambia, and Zimbabwe (PHIA surveys in these countries were conducted during 2015–2017).

** Information on HIV treatment regimen was not collected in the PHIA questionnaire, and some mothers might not have been on lifelong ART.

†† Method of consent was either oral or written depending on each country’s PHIA survey protocol.

§§ In Uganda, Geenius confirmation was not used. All specimens collected by venous blood draw were retested at the central laboratory using the national rapid testing algorithm used in the field.

¶¶ Option B+ was first introduced in Malawi in 2011 and expanded globally since 2013. “Treat-all” refers to ART initiation among all adults with HIV regardless of clinical stage and at any CD4 cell count. https://apps.who.int/iris/bitstream/handle/10665/186275/9789241509565_eng.pdf;jsessionid = 1461B0D372ABEF56426E1D7CD95CD354?sequence = 1.

*** Births included in this analysis occurred during 2012–2017. National HIV programs scaled up Option B+ in 2011, and adult treat-all approach was initiated in 2016.

††† The Accelerated Childen’s HIV/AIDS Treatment initiative was a 2-year program to double the number of children receiving ART in sub-Saharan Africa. https://journals.lww.com/jaids/Fulltext/2018/08152/Sustainability_and_Accelerating_Children_s.12.aspx.

§§§ Corresponding to children who were aged <1 year at the time of the survey.

¶¶¶ The first two pillars of the World Health Organization’s comprehensive approach to PMTCT are preventing HIV infection and preventing unintended pregnancies. https://www.who.int/hiv/pub/mtct/strategic/en/.

**** In the pooled data, percentages of children born to mothers who were treated with ART before pregnancy and delivered in the 1, 2 and 3 years preceding the survey were 55.3%, 51.9% and 51.3%, respectively, indicating a modest increase in the proportion of mothers treated with ART before pregnancy over time.

†††† Infant mortality rates (deaths in children aged <1 year per 1,000 live births), irrespective of maternal HIV status, from the PHIA surveys and Demographic and Health Surveys (DHS), respectively, in the eight countries are as follows: Eswatini (0.9% and 8.5%), Lesotho (2.6% and 5.9%), Malawi (1.8% and 4.2%), Namibia (1.1% and 3.9%), Tanzania (1.6% and 4.3%), Uganda (1.5% and 6.4%), Zambia (1.7% and 4.5%), and Zimbabwe (1.5% and 5.0%). DHS methods for estimating child mortality are available at https://dhsprogram.com/Data/Guide-to-DHS-Statistics/Early_Childhood_Mortality.htm.
